# Moyamoya Syndrome Associated with Henoch-Schönlein Purpura

**Published:** 2016

**Authors:** Reza SHIARI, Seyed Mohamad Hossein TABATABAEI NODUSHAN, Mohamad Mahdi MOHEBBI, Parvaneh KARIMZADEH, Mohsen JAVADZADEH

**Affiliations:** 1 Department of Pediatric Rheumatology, Shahid Beheshti University of Medical Sciences, Mofid Children’s Hospital, Tehran, Iran; 2 Faculty of Medicine, Shahid Beheshti University of Medical Sciences, Tehran, Iran; 3 Pediatric Neurology Research Center, Shahid Beheshti University of Medical Sciences, Tehran, Iran; 4 Pediatric Neurology Department, Mofid Children’s Hospital, Faculty of Medicine, Shahid Beheshti University of Medical Sciences, Tehran, Iran

**Keywords:** Moyamoya syndrome, Henoch-Schönlein Purpura, Headache

## Abstract

Some reports have shown the association between Moyamoya syndrome and autoimmune diseases. Herewith, we present a 3.5 yr old girl with Henoch- Schönleinpurpura (HSP) who was treated with steroids because of sever colicky abdominal pain. However, central nervous system manifestations such as headache, ataxia and vision impairment developed during 6 months of her outpatient follow-up. More evaluation using MRA revealed intracranial stenosis of internal carotid artery and arterial collaterals that were in favor of Moyamoya syndrome. To our knowledge, this is the first report of Moyamoya syndrome following henoch-schönleinpurpura.

## Introduction

Moyamoya is known as a disease with unknown pathogenesis presenting with small collateral vessels distal to the blockage in the brain blood circulation ( 1). Moyamoya is mostly presenting by progressive stenosis of the terminal part of the internal carotid artery and its main branches ( 2, 3). The condition can be solely found in the brain vasculature, known as moyamoya disease (MMD) or in association with systemic disorders, which is moyamoya syndrome (MMS) ( 4). HenochSchonleinpurpura (HSP) is the most common vasculitis during childhood that involves small vessels in the skin (5).

The major clinical manifestations of HSP are purpuraor petechiae, abdominal pain, arthritis or arthralgia and renal involvement (5-7). The annual incidence of HSP is different in various countries and falls in the range of 6.1 to 70.3 per 105 children (5, 6, 8). HSP usually is a self-limited disease, which has a good prognosis especially when the significant renal involvement is absent (8). The etiology of HSP is not clearly identified. Infections, vaccination, drugs and insect bite are considered as triggers of HSP (6-9). Although the pathogenesis of HSP is not clear, immunoglobulin A (Ig A) have a strong role in the pathogenesis of this disease (8, 10). 

Rarely HSP patients present signs of CNS or peripheral nervous system dysfunction ( 5, 11). Neurologic signs and symptoms can be reversible and/or irreversible and central nervous system involvement is reported in 1%–8% of children and are due to hypertension, renal failure or vasculitis ( 11, 12).

There are some reports showing the association between the moyamoya syndrome and autoimmune disorders such as Behcet’s disease, Sjögren’s syndrome, systemic lupus erythematosus and Graves disease (13). However, we could not find Moyamoya syndrome associated with HSP.

Here with, we present a 3.5 yr old Iranian girl diagnosed as having HSP and her fallow up showed the CNS involvement signs, an association with Moyamoya syndrome which has not been reported up to date. 

## Case report

In July 2013, a 3.5 yr old girl with history of gastroenteritis from 2 weeks earlier was taken to Mofid Children Hospital, Tehran, Iran, presenting with petechial and purpura on the lower limbs and buttock, ecchymosis on the right ear, edema in ankles, right wrist and right elbow, arthralgia and abdominal pain. She had neither any specific sign of respiratory or urinary tract infection, palpable lymphadenopathy nor any known underlying disease with similar manifestations. She had a surgery to treat her PDA one year ago.

Informed consent was taken for the patient’s parents. Due to the severe alternating abdominal pain, the patient was admitted to the ward and after physical examinations the routine and specific diagnostic laboratory tests were done and the results are as followings: White blood cells: 8110/mm3, platelet: 493000/mm3, hemoglobin: 11 g/ dL, C3: 114mg/dL (90-180), C4: 27.3mg/dL (10-40), CH50: 116% (51%-150%), A.N.A titer<1/40 (normal), C-reactive protein level: 27 mg/L (<10), Cr: 0.6 mg/dl, BUN: 12mmol/L. Liver function tests including SGOT and SGPT were within normal limits. Antistreptolysin O titer and throat culture for Streptococcus pyogenes (Group A β-hemolytic Streptococcus) were negative and urine analysis did not show any abnormal findings. 

Based on the examinations done by the medical staff the patient was finally diagnosed as having HSP. She was treated by steroid because of persistent colicky abdominal pain that dramatically responded. Finally, she discharged after complete recuperation. The patient was visited for 2, 4, and 6 weeks after discharging from hospital at the outpatient clinic. Her laboratory tests were normal but she expressed symptoms of headache during this period of follow-up.

Almost 4.5 month after discharging, she complicated by blurred vision and ataxia beside the headache she had. 

**Fig 1:**
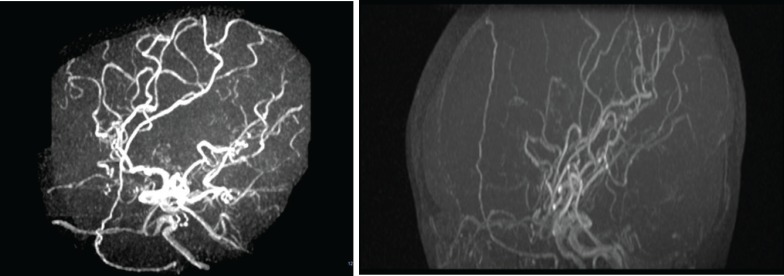
MRA image in sagittal projection (A and B) reveals stenosis of distal internal carotid arteries associated with collateral vessel formation

Admitting to the Children Medical Center, magnetic resonance angiography (MRA) was done to evaluate her CNS manifestations. The MRA showed internal carotid artery stenosis and diagnostic moyamoya view made by the small collateral vessels in the brain imaging defining the third grade of moyamoya according to Suzuki angiographic classification grades (Figure 1). 

The patient did not go under any brain surgery but was ordered to take oral antiplatelet (aspirin) daily however she has been suffering from intermittent headaches while on medication, as it is mentioned in the literature that moyamoya subsequent headache is refractory to the medical therapies ( 13). She has been under medical supervision for a period of more than 2 years up to now as outpatient follow-up. 

## Discussion

Moyamoya is an abnormal condition of cerebral arteries, mostly seen in Asia ( 15) and occurs in women twice as in men ( 16). Some genes are introduced to cause the disease but presenting the disease in only one of the identical twins strongly suggests the role of environmental factors in developing the syndrome ( 17). 

Although the exact process leading to moyamoya is still unknown but some probable conditions are most likely. 

MMS like cephalic or neck irradiation, skull base tumor, atherosclerosis of skull base arteries, chronic meningitis (especially tuberculosis meningitis), cerebral vasculitis, autoimmune angiitis, prothrombotic disorders and sickle cell disease ( 13).

In this article, we reported a 3.5 yr old Iranian girl who showed cerebral signs of moyamoya secondary to her inactive HSP. The time interval between primary disease and developing CNS complications is in a range from one week to 10 yr in the reported cases of systemic lupus erythematosus ( 13). In our case, there was 6 months interval between presentation of HSP and subsequent MMS. Natural history in MMS patients differs greatly and can be in a range with slowly progression or rapid progression with fulminant manifestations ( 19). The signs also depend on the vascular involvement pattern and area of the brain affected, but headache is one of the frequent complications, which has the quality like the migraine headaches ( 13). Vision impairment is also another complaint ( 20) which together with previous one was presented clearly in our patient.

None of the treatment protocols is able to postpone the process of the disease to its primary form and the plans are mostly to prevent catastrophic complications such as stroke ( 21). The long-term medical treatment mainly consists of aspirin to make the blood circulation better to the compromised areas of the brain ( 22). Some different surgical procedures are also used to help the patient with revascularization but the treatment plan mostly depends on the patient age and the process of the disease ( 23). 

Although there is not a definite plan to screen the people to find MMD cases but it is suggested to use diagnostic MRA to evaluate the patients presenting cerebral signs specially with an either active or inactive autoimmune disorder, positive family history of MMD in first degree relatives and other reported conditions such as Down’s syndrome or sickle cell anemia. In conclusion, here we described the association between autoimmune diseases and moyamoya syndrome and presented a case who developed MMS with inactive HSP after 6 months of the primary disease onset.
